# New Trends in Bioremediation Technologies Toward Environment-Friendly Society: A Mini-Review

**DOI:** 10.3389/fbioe.2021.666858

**Published:** 2021-08-02

**Authors:** Kunal Dutta, Sergey Shityakov, Ibrahim Khalifa

**Affiliations:** ^1^Department of Human Physiology, Vidyasagar University, Medinipur, India; ^2^Department of Chemoinformatics, Infochemistry Scientific Center, Saint Petersburg National Research University of Information Technologies, Mechanics and Optics (ITMO University), Saint-Petersburg, Russia; ^3^Food Technology Department, Faculty of Agriculture, Benha University, Moshtohor, Egypt

**Keywords:** environment, crude oils, greenhouse gases, polycyclic aromatic hydrocarbons, enzymes, machine learning, metabolic engineering

## Abstract

Today's environmental balance has been compromised by the unreasonable and sometimes dangerous actions committed by humans to maintain their dominance over the Earth's natural resources. As a result, oceans are contaminated by the different types of plastic trash, crude oil coming from mismanagement of transporting ships spilling it in the water, and air pollution due to increasing production of greenhouse gases, such as CO_2_ and CH_4_ *etc*., into the atmosphere. The lands, agricultural fields, and groundwater are also contaminated by the infamous chemicals *viz.*, polycyclic aromatic hydrocarbons, pyrethroids pesticides, bisphenol-A, and dioxanes. Therefore, bioremediation might function as a convenient alternative to restore a clean environment. However, at present, the majority of bioremediation reports are limited to the natural capabilities of microbial enzymes. Synthetic biology with uncompromised supervision of ethical standards could help to outsmart nature's engineering, such as the CETCH cycle for improved CO_2_ fixation. Additionally, a blend of synthetic biology with machine learning algorithms could expand the possibilities of bioengineering. This review summarized current state-of-the-art knowledge of the data-assisted enzyme redesigning to actively promote new research on important enzymes to ameliorate the environment.

## Graphical Abstract

Redesigned enzyme for bioremediation. 
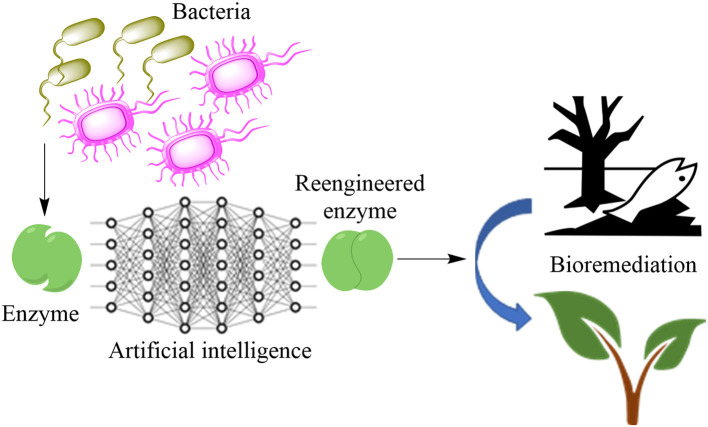

## Introduction

The present growth and development of modern human societies are sustained by the stability of the Holocene climate (Revell, 2020). However, the invariability of the stable Holocene climate had been overwhelmingly abused by unrestrained consumption without genuine attention to the environment. Moreover, due to such negligence, the total wilderness of the earth has been dramatically reduced to only 35% of what it once was (Revell, 2020). Global warming (Change, 2018), polar ice meltdown (Hansen et al., 2015), reduction of biodiversity (Underwood et al., 2009; Handa et al., 2014; Delgado-Baquerizo et al., 2020), and extinctions of important wild-life species (Thomas et al., 2004) are influencers in the global climate change. Our environment is not only ruined but also destroyed by human activities. Now, we must reverse the process (Revell, 2020). In addition, according to a recent report, corals are dying as a result of high ocean-water pH (Hoegh-Guldberg et al., 2017). However, corals are crucial for underwater biodiversity (Wagner et al., 2020). Moreover, ocean pollution is increasing due to plastic articles, crude oils (Price et al., 2003), *etc*., which are not coral-friendly (Monteiro et al., 2018; Forrest et al., 2019). However, plastic pollution has been identified (Xanthos and Walker, 2017), and alternative materials such as bioplastics (Peelman et al., 2013; Ashter, 2016; Brodin et al., 2017) and plant-based materials are replacing non-biodegradable plastics (Ashori, 2008; Mooney, 2009; Su et al., 2018). Besides, the termination of non-biodegradable plastic production is possible by strict government restrictions (Xanthos and Walker, 2017). Conversely, we are still very dependent on hydrocarbon oil (Holdren, 2006). Thus, disasters like oil spills in the middle of oceans have become common events (Magris and Giarrizzo, 2020). For example, the artic oil spill released about 21,000 tons of diesel into rivers and subsoil from a fuel tank near Norilsk, Russian Federation (Reuters, June 9, 2020).

**Figure 1:**
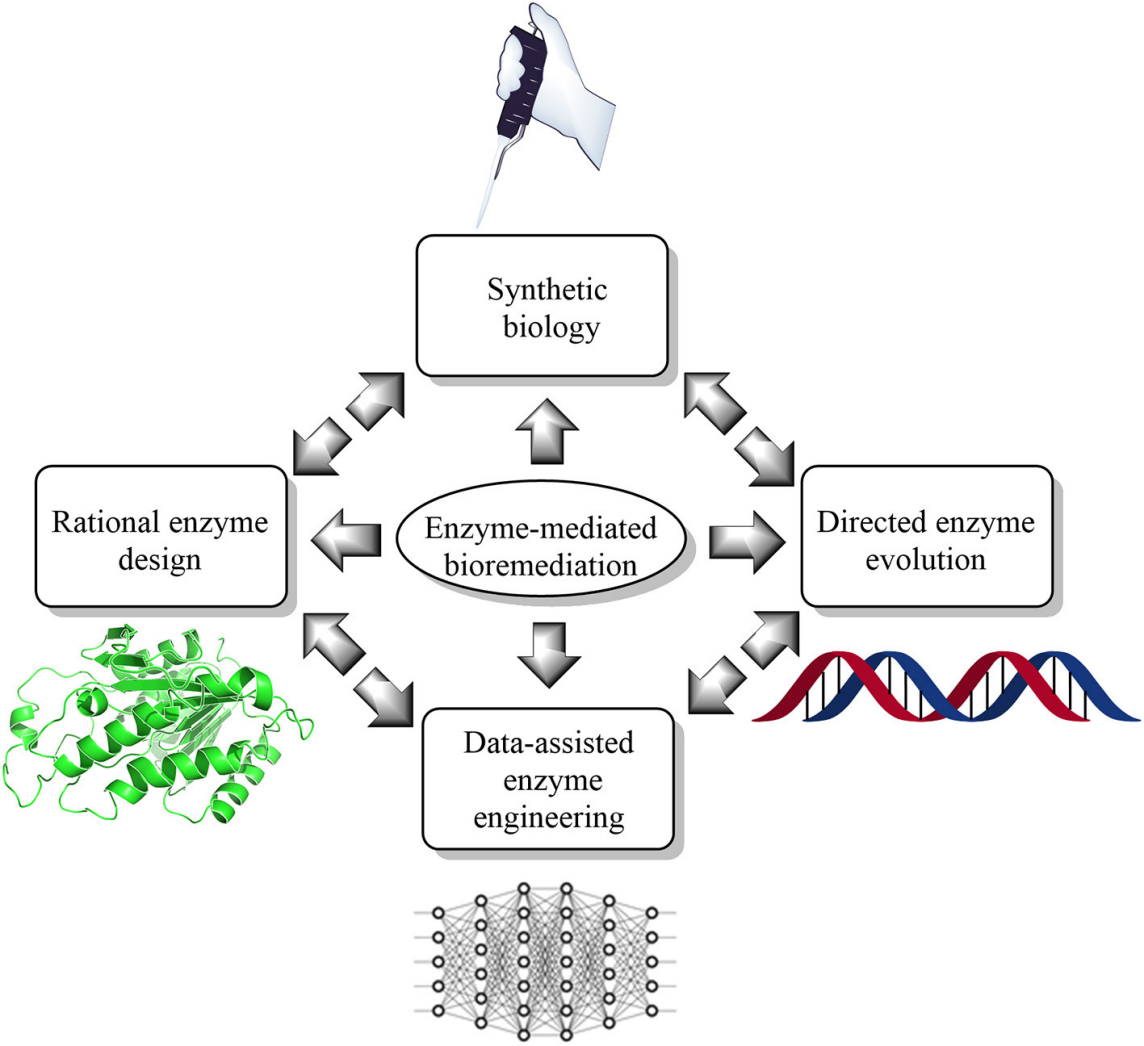
Current state-of-the-art trends and technologies for enzyme-mediated bioremediation, which include synthetic biology (enzyme reengineering and redesigning *in vitro*), rational enzyme design (molecular modeling and simulation), directed enzyme evolution (enhanced enzyme selection), and data-assisted enzyme engineering [artificial intelligence (AI) and machine learning (ML)].

Furthermore, the air we breathe is also not very healthy (Koenig, 2000; Carvalho, 2016; West et al., 2016). According to a recent report, the air quality index (AQI) in some cities are in critical condition (Chelani et al., 2002; Kumar and Goyal, 2011). Besides, the emission of high amounts of greenhouse gases such as CO_2_ and methane has also threatened the respiratory health of humans and animals (Marrero, 2010; Li S. et al., 2018). Additionally, soil finds itself to not be an exception to this list. Soil, as well as groundwater, is contaminated by notorious chemicals such as polycyclic aromatic hydrocarbons (PAH), pyrethroids pesticides (Holmes et al., 2008; Deng et al., 2020), bisphenol-A, and dioxanes *etc.*, (Lee and Peart, 2000; Haritash and Kaushik, 2009). Bioremediation is one way to restore our environment from devastating damage (Vidali, 2001). Besides, bioremediation is an environment-friendly approach that uses the microbial enzyme to metabolize the pollutant as a nutrient for microbes (Vidali, 2001). For example, of bioremediation by enzyme engineering, which can improve the function of the microbial enzyme (Ali et al., 2020) by means of directed evolution (Kuchner and Arnold, 1997) and rational (Cedrone et al., 2000) and semi-rational approaches (Lutz, 2010). However, engineering enzymes in the data-assisted synthetic biology landscape could accelerate the hunt of the “super-enzyme” in environmental perspectives. However, as this is a new frontier to the scientific literature body, only a handful of the kindest efforts are available at present (Ajjolli Nagaraja et al., 2020; Lawson et al., 2020; Mou et al., 2020; Robinson et al., 2020; Siedhoff et al., 2020; Wittmann et al., 2020). Herein, we have summarized current state-of-the-art knowledge of the data-assisted enzyme redesigning (Figure 1) to promote new studies on enzyme redesigning from an environmental perspective.

## Scientific Background of Enzyme Redesigning

### Directed Evolution (DE) of Enzymes

Enzyme redesigning by “directed evolution” was introduced in 1997 (Kuchner and Arnold, 1997). In that same year, 40x optimization of arsenic resistance property was reported (Crameri et al., 1997). After that, many research groups reported the DE of enzymes because it is a novel approach to redesign biocatalyst (Kuchner and Arnold, 1997), (MacBeath et al., 1998). For example, directed evolution is successful in improving different enzymes, *viz., Staphylococcal nuclease* (100-fold) (Pedersen et al., 1998), an efficient RNA polymerase (Xia et al., 2002), a Cre recombinase (Santoro and Schultz, 2002), a new enzyme function by synthetic transformation (Turner, 2009), or enzymes with industrial values (Zhao et al., 2002; Cherry and Fidantsef, 2003; Eijsink et al., 2005; Liang et al., 2014; Porter et al., 2016). The biosynthetic pathway is also completely manipulated by enzyme redesigning (Johannes and Zhao, 2006). Besides, directed evolution methods were progressively improved with time (Arnold and Georgiou, 2003; Lutz and Patrick, 2004; Leemhuis et al., 2009). Table 1 summarizes enzyme engineering approaches and new trends, that could improve enzyme-mediated bioremediation toward an environment-friendly society.

**Table 1:** Summary of enzyme engineering approaches and new trends.

**Type of improvements**	**Brief about the advancements**
Workflow/pipeline: for DE.	A machine learning assisted directed evolution (MLDE) workflow. It tests many design considerations of the MLDE pipeline (Wittmann et al., 2020). Available at https://github.com/fhalab/MLDE
A predictive DE method.	An innovative sequence–activity relationship (innov'SAR) method. This method combines wet-lab experimentation and computational protein design. An epoxide hydrolase from *Aspergillus niger* is used in this model (Cadet et al., 2018).
A predictive model for catalytic turnover number (*k*_cat_).	The model has identified a diverse set of enzyme features, for example, structure, biochemistry, and the network. These networks are applicable for *in vivo* and *in vitro* enzyme turnover rates. Finally, the predicted catalytic turnover rates are correlated with experimental results (Heckmann et al., 2018).
A predictive model for optimal growth temperature and catalytic temperature optima (*T_*opt*_*).	This model is used to generate the optimal catalytic temperature of the enzyme. It helps to redesign enzymes for performance at extreme temperatures (Li et al., 2019).
A predictive model of concentration for metabolic flux optimization.	The model uses the artificial neural network. It is helpful for the optimization of *in silico* enzyme concentration prediction. The accurate enzyme concentration is helpful for the cell-free enzyme assay (Ajjolli Nagaraja et al., 2020).
Machine learning (ML) sequence function models.	It provides steps for machine learning sequence function-based models. This model is helpful for accurate protein engineering through DE (Yang et al., 2019).
ML-based improvement of proteinase K.	This model uses two cycles of machine learning algorithms. The catalytic efficiency of the enzyme improves about 20x by this strategy. The significant advantages of this model are that it tests only 95 variants of redesigned proteinase K (Liao et al., 2007).
Supervised machine learning-based ligand affinity, predation models.	This work provides detail information on the supervised machine learning-based model. This model predicts the ligand affinity of the enzymes (S Heck et al., 2017).
An ensemble learning model for accurate prediction of the optimum catalytic temperature (*T_*opt*_*) of the enzymes.	It is an improved ensemble learning model. This model eliminates error in the temperature range prediction of the enzyme (Gado et al., 2020).
ML-based prediction model for enzyme activity and substrate specificity of thiol superfamily enzyme.	It is a model of thiolase superfamily enzyme. It measured the activity of 73 diverse bacterial thiolase (Robinson et al., 2020). Available at https://github.com/serina-robinson/thiolase-machine-learning/
A high-quality and high-throughput deep learning (DL) model for accurate enzyme commission (EC) number prediction model.	It is a high-precise deep learning model. It uses three convolutional neural networks and homology analysis. This model is useful for Enzyme Commission (EC) number prediction (Ryu et al., 2019).
A multi-level machine learning model enzyme-substrate prediction.	It applies experimental enzyme activity data, structure, ligand docking, and physiochemical properties. This model is based on a bacterial nitrilase (Mou et al., 2020).
A multi-level hierarchical deep learning model for multi-functional enzyme prediction.	This deep learning model is based on a novel loss of function. This loss of function is associated with the relationship between different levels and self-adapted level assigning threshold (Zou et al., 2019).
The proposed machine learning model for class selective optimization of enzyme.	This work emphasized the application of machine learning. It also discussed the practical improvement of biotechnology, metabolic engineering, and synthetic biology (Ng, 2020).
DE model of the enzyme based on a statistical exploration of sequence-function space.	This report provides the usefulness of machine learning assisted directed evolution. It highlights the disadvantages of random mutagenesis, DNA shuffling, *etc*. (Fox and Huisman, 2008).
Automatic single, multi-level enzymatic function prediction model.	It is an accurate EC number prediction model. The model combines both structure and amino acid sequence information. This approach also includes feature level and decision level investigation (Amidi et al., 2017). This machine learning model is available at https://figshare.com/s/a63e0bafa9b71fc7cbd7
A ML model for identification of the reactivity promoting region (RPR) of the enzyme.	This model uses multiples descriptors. The descriptors are substrate conformation, metal coordinate geometry, and substrate bond polarization. This model promotes the substrate reactivity with <85% accuracy (Bonk et al., 2019).
A Random Forest-based machine learning model for enzyme reaction prediction.	This model predicts EC number by two-fold accuracy optimizations. This prediction optimization is achieved by sequence data and enzyme-substrate models (Watanabe et al., 2020).
A supported vector machine (SVM) model for substrate specificity prediction.	This SVM model uses a large set of data. Moreover, it is 80% accurate with 30% (approx.) less compound in the datasets (Pertusi et al., 2017).
A quantitatively validated machine learning model for enzymatic pathway prediction.	This ML model uses an extensive data set of 123 biochemical pathways. Moreover, the decision tree, logistic regression, etc. are used as an input (Dale et al., 2010).
A multi-level machine learning model for prediction of the enzymatic mechanism.	The model utilizes a large set of databases, for example, InterPro, Catalytic site Atlas, MACiE, EzCatDb, and SFLD. It also uses off-the-shelf K-Nearest Neighbors multi-label algorithm (De Ferrari and Mitchell, 2014). Available online at http://sourceforge.net/projects/ml2db/
A high-performance ML-based tool for metabolic pathway prediction of plant enzymes.	This model uses sequence similarities of the enzymes with the reference sequence. It is also available for local installation using a Graphical user interface (de Oliveira Almeida and Valente, 2020).
A hyper network model for enzymatic weight update.	The molecular algorithm is based on training data and targets internal loop structures in DNA and ensemble learning (Baek et al., 2019).
Multiple machine learning algorithms for prediction enzymatic reactions.	This algorithm uses three reaction fingerprints and seven ML models. This model can predict the enzymatic reactions catalyzed by oxidoreductase and hydrolase (Cai et al., 2018).
Supervised machine learning-based enzyme class prediction.	This model uses amino acid sequence-derived features. These features are amino acid composition, dipeptide composition, amino acid distribution, *etc*. Besides, support vector machine recursive feature elimination and Random Forest are also used by this model (Yadav and Tiwari, 2015).
An online server for enzyme selective pathway design.	“Selenzyme” is an assembled tool with the extended application of many tools such as machine learning, antiSMASH, *etc*. (Carbonell et al., 2018). Available at http://selenzyme.synbiochem.co.uk/
A semisupervised Gaussian model for enzyme search and Michaelis—Menten constant *K_*m*_* prediction.	This automatic semi-supervised Gaussian model uses chemical transformation fundamentals to provide probability estimates. Moreover, the probability estimate model is confirmed in *E. coli* (Mellor et al., 2016).
Machine learning models for metabolic engineering.	This work illustrates how machine learning models can overcome the rate-limiting step and optimize complex metabolic networks (Zhou et al., 2020).
A deep learning model for accurate enzyme function prediction.	DEEPre is a deep learning model based on accurate prediction of EC number (Li Y. et al., 2018). Available at http://www.cbrc.kaust.edu.sa/DEEPre
A machine learning-based web-server for prediction of the enzyme class.	SMV-Prot prediction model is based on protein sequences irrespective of the similarities and available at http://bidd2.nus.edu.sg/cgi-bin/svmprot/svmprot.cgi (Li et al., 2016).

### Rational Approach of Enzyme Redesigning

Rational and semi-rational approaches of the enzyme redesigning are supervised by high-end computation power for the mutant library preparation (Cedrone et al., 2000). Conversely, in directed evolution, a random mutation library is prepared without computation power (Kuchner and Arnold, 1997). Therefore, in DE, a random mutation library preparation is more tedious and time-consuming. However, active learning, machine learning, and deep learning-assisted enzyme redesigning are state-of-the-art methods for enzyme redesigning. Since the year 2013, machine learning has become popular in studying science and engineering (Zhang et al., 2020). Furthermore, in the late 20^th^ century, computational technologies contribute to data-assisted enzyme engineering (Cedrone et al., 2000; Chen, 2001; Lutz, 2010; Otten et al., 2010; Steiner and Schwab, 2012). For example, successful examples of the rational and combinatorial approaches of enzyme redesigning are as follows: site-directed mutagenesis with a combinatorial approach (Cedrone et al., 2000), a structure-based improvement of the non-ribosomal peptide synthetase (Chen, 2001), active site redesigning (Toscano et al., 2007), enantioselectivity-based improvement (Otten et al., 2010), site-directed saturation mutation analysis (Schneider et al., 2010), *de novo* substrate-based enzyme engineering (Steiner and Schwab, 2012), a combinatorial approach for improving alcohol dehydrogenase (Zhang et al., 2015), the rational-designed dual active site of a protein scaffold (Shu et al., 2016), 100x optimization of a selenoenzyme (Wang et al., 2018), heavy enzyme redesigning (Scott et al., 2019), 40x catalytic and 39x stereoselectivity enhancement of a decarboxylase (Payer et al., 2018), and widening active site tunnel by backbone redesigning (Rigoldi et al., 2020), etc.

## Data-Assisted Enzyme Engineering (DAEE)

Studies on the structure–function relationship of enzymes are possible with the help of the latest biophysical tools. Moreover, the protein database (PDB) (Sussman et al., 1998) and similar databases (El-Gebali et al., 2019) offer excellent opportunities for a data scientist to analyze and optimize particular enzyme structures from a large volume of data. On the other hand, machine learning (Mazurenko et al., 2019) and deep learning are two approaches where specific algorithms are needed (LeCun et al., 2015). So far, there are limited numbers of research studies available in this new field of enzyme engineering.

## Synthetic Biology and Data-Assisted Enzyme Engineering

Synthetic biology offers the possibility to redesign the chemical composition of biological molecules. It can also engineer natural DNA polymerase to catalyze a new type of genetic material called Xeno nucleic acids (XNA) (Glasscock et al., 2016). On the other hand, machine learning offers excellent advantages to handle big data. Thus, analyzing big data with machine learning provides new insights to improve the enzyme (Mazurenko et al., 2019). Besides, the synthetic biology industry or syndustry is a growing area of the bioeconomy (Bueso and Tangney, 2017), and it includes a wide range of enzyme applications. In comparison to some standard techniques of CO_2_ fixation including cell-free synthetic biology and ultrahigh-throughput enzyme engineering approaches using omics-based big data, the CETCH cycle was designed to be 3x faster, providing more possibilities for mutant library generation and screening (Young and Alper, 2010 Schwander et al., 2016; Quaglia et al., 2017; Badenhorst and Bornscheuer, 2018; García-Granados et al., 2019; Jiang et al., 2020).

## Data-Assisted Synthetic Biology and Bioremediation

In the literature, the majority of bioremediation reports are about the natural capacity of the microbial enzymes. However, the natural enzymatic efficiency is slow compared to any redesigned or “tailor-made” enzymes (Schwander et al., 2016). Also, a microorganism has to follow a very long route to reach the final TCA cycle (Dutta et al., 2018). Yet, bioremediation with the data-assisted synthetic biology is overlooked. Conversely, bioremediation properties/pathways of a natural enzyme could be optimized by data-assisted assisted enzyme engineering.

## Air Pollution, CO_2_ Fixation, and Ribulose-1,5-Bisphosphate Carboxylase/Oxygenase (RUBisCO)

Air pollution due to greenhouse gases is a significant problem for public health (Costello et al., 2009; Bierwirth, 2018) as well as on the atmosphere and climate change (Costello et al., 2009; Ramanathan and Feng, 2009; El Zein and Chehayeb, 2015). Ribulose-1,5-bisphosphate carboxylase-oxygenase (RuBisCO) is a primary enzyme that catalyzes CO_2_ fixation in photosynthetic plants (Hatch and Slack, 1970). As plants hold great potential to reduce air pollution (Chung et al., 2011), redesigning RuBisCO could be an ideal target for ecology and environmental protection. The structure of RuBisCO varies with the plant species, and some RuBisCO variants are also available in the PDB database. Yet, studies on the identification of vital amino acids are still sparse. But, Ile-165 and Met-331 mutations of RuBisCO in *Rhodospirillum rubrum* might alter the enzyme function. Besides, the Ala-47 mutation at the C-terminus near the active site significantly improves the carboxylation efficiency of RuBisCO.

Furthermore, some “form-I and -III” mutations (*Rhodospirillum rubum*) in the C-terminus have resulted in the loss of the enzyme activity (Satagopan et al., 2014). Moreover, nitrosylation is crucial for RuBisCO activation in Galdieria sulphuraria, which has been overlooked for many years (Stec, 2012). Additionally, Mg^2+^ and few amino acids serve a vital role in the activation and carbamoylation process of RuBisCO (Okano et al., 2002). These results are encouraging to optimize RuBisCO by data-assisted enzyme engineering.

## Ocean Water Oil Spill, Bioremediation, and Methane Monooxygenase (MMO)

Modern human society depends on petroleum hydrocarbons. However, human activities on the oil spill and hydrocarbon pollution occur in many parts of the world, especially in the middle-east (Elsayed and Ammar, 2020; Nwachukwu et al., 2020; Wang D. et al., 2020). Bacterial enzymes metabolize crude oil fractions containing hydrocarbons (Stauffer et al., 2008). For example, methane monooxygenase (MMO), commonly found in methanotrophic bacteria (Singh and Singh, 2017), could be a perfect target for data-assisted enzyme engineering to improve oil-bioremediation strategy. MMOs are of two types, *i.e*., soluble methane monooxygenase (sMMO) and particulate methane monooxygenase (pMMO) (Lipscomb, 1994). The structural features of sMMO are previously discussed in a more detailed manner (Banerjee et al., 2019). The sMMO active site is mainly composed of E144, H147, E209, E243, and H246 residues. Similarly, Culpepper et al. have characterized the molecular structure of pMMO (Culpepper and Rosenzweig, 2012), and Rigoldi et al. have shown improved catalytic efficiency of pMMO improved by widening the diameter of the active site (Rigoldi et al., 2020). A recent study on sMMO showed an essential role of O_2_ transport passage to the active site termed as W308-tunnel (Jones et al., 2020). Thus, reengineering sMMO might improve the enzymatic efficiency.

## Soil and Groundwater Contamination By Pahs and Aromatic Ring Hydroxylating Dioxygenase (ARHD)

The polycyclic aromatic hydrocarbon is a harmful chemical comprising 16 variants (PAHs) added to the priority list by the US Environmental Protection Agency (Andersson and Achten, 2015; Zelinkova and Wenzl, 2015; Dutta et al., 2017, 2018). These PAHs' physiochemical and toxic properties raise a significant concern over their impact on soil and groundwater contamination (Wang et al., 2009). Moreover, several reports showed evidence of PAH contamination in different soil and groundwater sources (Sushkova et al., 2018; Haleyur et al., 2019; Lu et al., 2019; Liang et al., 2020; Pacwa-Płociniczak et al., 2020; Picariello et al., 2020; Wang Y. et al., 2020; Wolf et al., 2020; Ambade et al., 2021a,b; Qiao et al., 2021). Therefore, the PAH contamination problem requires more research in this direction using chemical cleavage. PAHs are composed of two or more fused aromatic rings (Haritash and Kaushik, 2009), which can cleave by the aromatic ring hydroxylating dioxygenase, estradiol ring cleavage dioxygenase, and estradiol ring cleavage dioxygenase (Arora et al., 2009). Aromatic ring hydroxylating dioxygenase (ARHD) is a promising enzyme for this purpose, composed of an iron–sulfur flavoprotein and an iron–sulfur ferredoxin subunit (Butler and Mason, 1996). The advantage of this enzyme is that it can catalyze biodegradation of more than one PAH species and initiates the degradation of 44 different aromatic compounds (Parales and Resnick, 2006). Therefore, this enzyme might be a promising tool to implement in environmental applications (Tan and Parales, 2016), and its further reengineering using a data-assisted enzyme engineering approach could be advantageous.

## Conclusion

In this condensed review, we have identified different approaches of data-assisted enzyme engineering that could be applied on RuBisCO for air pollution, methene monooxygenase for crude-oil bioremediation, and aromatic ring hydroxylating dioxygenase for bioremediation of PAHs from soil and groundwater. Future directions can be referred to a design and development of the pipelines, algorithms, and protocols, integrating aforementioned state-of-the-art technologies for enzyme-mediated bioremediation, such as synthetic biology, rational enzyme design, directed enzyme evolution, and AI/ML-assisted enzyme engineering. Overall, this review might help to potentiate more research on this direction, which is an urgent need in this present environmental crisis. However, challenges remain active to apply data-assisted synthetic biology in improving bioremediation, but with computation power and up-gradation of the coding skills, these could be overcome.

## Author Contributions

SS and KD conceptualized the topic. KD performed the scientific literature search and wrote the manuscript. SS proofread the manuscript. SS and IK wrote part of the manuscript. All co-authors read the manuscript. All authors contributed to the article and approved the submitted version.

## Conflict of Interest

The authors declare that the research was conducted in the absence of any commercial or financial relationships that could be construed as a potential conflict of interest.

## Publisher's Note

All claims expressed in this article are solely those of the authors and do not necessarily represent those of their affiliated organizations, or those of the publisher, the editors and the reviewers. Any product that may be evaluated in this article, or claim that may be made by its manufacturer, is not guaranteed or endorsed by the publisher.
